# Genome-enabled prediction using probabilistic neural network classifiers

**DOI:** 10.1186/s12864-016-2553-1

**Published:** 2016-03-09

**Authors:** Juan Manuel González-Camacho, José Crossa, Paulino Pérez-Rodríguez, Leonardo Ornella, Daniel Gianola

**Affiliations:** Colegio de Postgraduados, Campus Montecillo, Texcoco, México 056230 México; Biometrics and Statistics Unit (BSU), International Maize and Wheat Improvement Center (CIMMYT), Apdo Postal 6-641, México DF, 06600 24105 México; NIDERA SEMILLAS S.A., Ruta 8 Km. 376, 2600 Venado Tuerto, Argentina; Department of Animal Sciences, University of Wisconsin, Madison, 53706 USA

**Keywords:** Average precision, Bayesian classifier, Genomic selection, Machine-learning algorithm, Multi-layer perceptron, Non-parametric model

## Abstract

**Background:**

Multi-layer perceptron (MLP) and radial basis function neural networks (RBFNN) have been shown to be effective in genome-enabled prediction. Here, we evaluated and compared the classification performance of an MLP classifier versus that of a probabilistic neural network (PNN), to predict the probability of membership of one individual in a phenotypic class of interest, using genomic and phenotypic data as input variables. We used 16 maize and 17 wheat genomic and phenotypic datasets with different trait-environment combinations (sample sizes ranged from 290 to 300 individuals) with 1.4 k and 55 k SNP chips. Classifiers were tested using continuous traits that were categorized into three classes (upper, middle and lower) based on the empirical distribution of each trait, constructed on the basis of two percentiles (15–85 % and 30–70 %). We focused on the 15 and 30 % percentiles for the upper and lower classes for selecting the best individuals, as commonly done in genomic selection. Wheat datasets were also used with two classes. The criteria for assessing the predictive accuracy of the two classifiers were the area under the receiver operating characteristic curve (*AUC*) and the area under the precision-recall curve (*AUCpr*). Parameters of both classifiers were estimated by optimizing the *AUC* for a specific class of interest.

**Results:**

The *AUC* and *AUCpr* criteria provided enough evidence to conclude that PNN was more accurate than MLP for assigning maize and wheat lines to the correct upper, middle or lower class for the complex traits analyzed. Results for the wheat datasets with continuous traits split into two and three classes showed that the performance of PNN with three classes was higher than with two classes when classifying individuals into the upper and lower (15 or 30 %) categories.

**Conclusions:**

The PNN classifier outperformed the MLP classifier in all 33 (maize and wheat) datasets when using *AUC* and *AUCpr* for selecting individuals of a specific class. Use of PNN with Gaussian radial basis functions seems promising in genomic selection for identifying the best individuals. Categorizing continuous traits into three classes generally provided better classification than when using two classes, because classification accuracy improved when classes were balanced.

**Electronic supplementary material:**

The online version of this article (doi:10.1186/s12864-016-2553-1) contains supplementary material, which is available to authorized users.

## Background

Complex traits of economic importance in animal and plant breeding seem to be affected by many quantitative trait loci (QTL), each having a small effect, and are greatly influenced by the environment. Predicting these complex traits using information from dense molecular markers exploits linkage disequilibrium (LD) between molecular markers and QTL. Basically, genomic selection works by capturing realized relationships between individuals and, to an extent, by capturing the effects of QTL via their linkage or LD with markers. Genomic selection (GS) regression models use all available molecular marker and phenotypic data from an observed base (training population) to predict the genetic values of yet unphenotyped candidates for selection (testing population) whose marker genotypes are known.

There is a vast literature describing statistical methods that use different functional forms on markers for predicting genetic values, e.g., [1, 2], starting with the seminal work of [3], which proposed regressing phenotypes on all available markers using a Gaussian linear model with different prior distributions on marker effects. Several parametric and semi-parametric methods have been described and used thereafter for genome-enabled prediction in animals and plants [4–11].

The basic quantitative genetic model *y*_*i*_ = *g*_*i*_ + *γ*_*i*_ (*i* = 1, … *n* individuals) describes the *i*^*th*^ response or phenotype (*y*_*i*_) expressed as a deviation from some general mean (μ) as the sum of an unknown genetic value (*g*_*i*_) plus a model residual γ_*i*_. The unknown genetic value can be represented as a complex function of genotypes with a large number of genes. However, since the genes affecting a trait are unknown, this complex function can be approximated by a regression of phenotype on marker genotypes where a large number of markers {*x*_*i*1_, …, *x*_*ip*_} (*x*_*ij*_ is the number of copies of one of the two alleles observed in the *i*^*th*^ individual at the *j*^*th*^ marker) may be used as regressors for predicting the genetic value of the *i*^*th*^ individual. Thus, for *u*(**x**_*i*_) = *u*(*x*_*i*1_, … *x*_*ip*_), the basic model becomes *y*_*i*_ = *u*_*i*_ + *γ*_*i*_, where γ_*i*_ includes errors due to unspecified environmental effects, imperfect linkage disequilibrium between markers and the QTL affecting the trait, and unaccounted gene × gene and gene × environment interactions.

In several applications, *u*(**x**_***i***_) is a parametric linear regression with form *u*(*x*_*i*1_, … *x*_*ip*_) = ∑_*j* = 1_^*p*^ *x*_*ij*_*β*_*j*_,, where β_*j*_ is the substitution effect of the allele coded as ‘one’ at the *j*^*th*^ marker. The linear regression function becomes *y*_*i*_ = ∑_*j* = 1_^*p*^ *x*_*ij*_*β*_*j*_ + *γ*_*i*_. The regression function *u*(**x**_*i*_) can also be represented by semi-parametric models, such as reproducing kernel Hilbert space (RKHS) regressions or by different types of neural networks (NN) such as the multilayer perceptron or radial basis functions [5, 8, 11–14]. Several penalized linear regression models and Bayesian shrinkage estimation methods have been applied to genome-enabled prediction [1]. Similarly, regularized machine learning has been used for predicting complex traits [15]. Recently, two-layer feed-forward NN with backpropagation were implemented in various forms using German Fleckvieh and Holstein-Friesian bull data and high prediction accuracies were achieved [16]. Likewise, a multi-layer NN classifier was applied to study genetic diversity in simulated experiments [17].

Nonparametric classification models are a branch of supervised machine learning that has been successfully applied in several fields of knowledge, e.g., text mining, bioinformatics and genomics [18, 19]. Particularly in applied genomic breeding programs and depending on the trait under consideration, the objective of classification is to focus on candidates for selection contained in the upper or lower classes of the prediction space. A common classification problem arises when an input marker vector **x**_*i*_ ∈ *ℝ*^*p*^ is to be assigned to one of *S* classes by a classifier. The classifier is trained using a set of training pairs (**x**_*i*_, *c*_*i*_), (*i* = 1, … *n* individuals), where *c*_*i*_ describes the class label (*k*) to which **x**_*i*_ belongs, (*k* = 1 … *S*), where *S* represents the number of classes. Usually, *c*_*i*_ is transformed into a vector **c**_*i*_ of dimension *S* × 1, with 1 in class *k* and 0 otherwise.

The multi-layer perceptron (MLP) classifier is a typical architecture of feed-forward NN with at least a hidden layer and an output layer, where both layers have nonlinear and differentiable transfer functions. The nonlinear transfer function in the hidden layer enables an NN to act as a universal approximation method. The training process of an MLP for each individual *i*, with input vector **x**_*i*_ and target class **c**_*i*_, typically uses the error backpropagation learning algorithm [20]. This process requires a lot of computational time when the number of input variables is large.

The probabilistic neural network (PNN) was proposed by [21] and is widely used in pattern recognition and classification. PNN classifies an input vector **x**_*i*_ into a specific *k* class such that the specific class has the maximum probability of being a correct assignment. PNN provides an optimum pattern classifier that minimizes the expected risk of wrongly classifying an object, and is a very efficient (in terms of computational time) classification method. The PNN training algorithm is simpler and faster than that of the MLP approach because PNN parameters are estimated directly from the input data and an iterative procedure is not required. Further, PNN guarantees convergence to a Bayes classifier if enough training examples are provided [22]. Several classification methods such as support vector machines and random forests have been applied in GS [23–25]. However, despite the apparent advantages of PNN, no PNN classifiers have been applied in GS so far.

The objective of this research was to assess the performance of two NN classifiers, MLP and PNN (based on Gaussian kernels), to select individuals belonging to a specific class of interest (target class). In an applied GS context, the problem should be formulated according to whether the focus is on selecting individuals into the upper, middle or lower classes, depending on the trait under selection. Then the question is how many of the predicted individuals classified in the target class are actually observed in that class. The problem is posed as follows: given an input vector **x**_*i*_ of *p* markers for the *i*^*th*^ individual, each individual *i* in the testing set must be classified in a class of interest of the phenotypic response. Classes were defined considering different percentiles of the target trait, specifically, 15 and 30 % for the upper and lower classes were analyzed.

## Methods

This section has four parts: first the two datasets are described; second, the strategy for categorizing the datasets is explained; third, the multilayer perceptron neural network (MLP) and probabilistic neural network (PNN) are described, and finally, the criteria used to assess model accuracy for classifying the best individuals based on genomic information are described.

### Maize datasets

The maize datasets include 16 trait-environment combinations measured on 300 tropical lines genotyped with 55,000 SNPs each; these datasets were previously used by [8]. Four datasets contain information on the complex trait grain yield (GY) evaluated under severe drought stress (GY-SS) and well-watered conditions (GY-WW), and in high yielding (GY-HI) and low yielding (GY-LO) environments. Another six datasets include information on days to anthesis or male flowering (MFL), on days to silking or female flowering (FFL), and the MFL to FFL interval (ASI) evaluated under severe drought stress (SS) and well-watered (WW) environments. The remaining six datasets contain information on gray leaf spot (GLS) resistance evaluated in six CIMMYT international trials (GLS-1 to GLS-6). The number of individuals and the type and number of markers are presented in Table 1; for further details, see [8].

**Table 1:** Maize datasets – three classes

				Number of individuals		Number of individuals		Number of individuals	
Data set	Trait-environment combination	Number of SNP markers	Total number of individuals	Upper	Upper	Middle	Middle	Lower	Lower
				15 %	30 %	40 %	70 %	15 %	30 %
GY-HI	Yield in high yielding environment	46374	267	40	80	107	187	40	80
GY-LO	Yield in low yielding environment	46374	269	40	81	107	189	40	81
GY-WW	Yield in well watered	46374	242	36	73	96	170	36	73
GY-SS	Yield in drought stressed	46374	242	36	73	96	170	36	73
ASI-WW	Anthesis-silking interval in well watered	46374	258	39	79	102	180	39	77
ASI-SS	Anthesis-silking interval in drought stressed	46374	258	40	77	103	179	39	78
MFL-WW	Male flowering time in well watered	46374	258	40	139	103	178	40	78
MFL-SS	Male flowering time in drought stressed	46374	258	39	77	104	179	40	77
FFL-WW	Female flowering time in well watered	46374	258	39	77	104	179	40	77
FFL-SS	Female flowering time in drought stressed	46374	258	39	77	104	180	39	77
GLS-1	Gray leaf spot in environment 1	46374	272	42	87	68	170	60	117
GLS-2	Gray leaf spot in environment 2	46374	280	48	85	77	176	56	118
GLS-3	Gray leaf spot in environment 3	46374	278	47	85	107	168	63	86
GLS-4	Gray leaf spot in environment 4	46374	261	48	96	74	154	59	91
GLS-5	Gray leaf spot in environment 5	46374	279	48	97	84	188	43	98
GLS-6	Gray leaf spot in environment 6	46374	281	63	85	90	140	78	106

Trait–environment combination, number of markers, total number of individuals, number of individuals in the upper 15 and 30 % classes, in the middle 40 and 70 % classes, and in the lower 15 and 30 % classes from the empirical cumulative distribution function

### Wheat datasets

These datasets include 306 wheat lines from the CIMMYT Global Wheat Program (GWP) that were genotyped with 1717 Diversity Array Technology (DArT) markers generated by Triticarte Pty. Ltd. (Canberra, Australia; http://www.diversityarrays.com), which is a whole-genome profiling service laboratory. Two traits were analyzed, grain yield (GY) and days to heading (DTH), which were evaluated in different environments (year-drought stress-agronomic treatments). GY was measured in seven environments and DTH in ten environments. The number of individuals and the type and number of markers are presented in Table 2; for further details, see [11].

**Table 2:** Wheat datasets – three classes

						Number of individuals		Number of individuals		Number of individuals	
Data set	Agronomic management	Site in Mexico	Year	Number of SNP markers	Total number of individuals	Upper	Upper	Middle	Middle	Lower	Lower
						15 %	30 %	40 %	70 %	15 %	30 %
GY-1	Drought-bed	Cd. Obregon	2009	1717	306	46	92	119	211	49	95
GY-2	Drought-bed	Cd. Obregon	2010	1717	306	47	92	122	213	46	92
GY-3	Drought-flat	Cd. Obregon	2010	1717	263	39	80	104	185	39	79
GY-4	Full irrigation-bed	Cd. Obregon	2009	1717	304	46	92	120	212	46	92
GY-5	Full irrigation-bed	Cd. Obregon	2010	1717	306	46	94	118	214	46	94
GY-6	Heat-bed	Cd. Obregon	2010	1717	306	46	94	120	214	46	92
GY-7	Full irrigation-flat	Cd. Obregon	2010	1717	263	39	79	105	185	39	79
DTH-1	Drought-bed	Cd. Obregon	2009	1717	306	53	100	93	197	56	113
DTH-2	Drought-bed	Cd. Obregon	2010	1717	306	50	93	117	198	58	96
DTH-3	Drought-flat	Cd. Obregon	2010	1717	263	40	86	77	177	46	100
DTH-4	Full irrigation-bed	Cd. Obregon	2009	1717	306	59	107	107	173	74	92
DTH-5	Full irrigation-bed	Cd. Obregon	2010	1717	306	47	101	105	207	52	100
DTH-6	Toluca	Toluca	2009	1717	306	122	122	75	93	91	109
DTH-7	El Batan	El Batan	2009	1717	306	66	104	101	175	65	101
DTH-8	Small observation plot	Cd. Obregon	2009	1717	301	58	101	100	182	61	100
DTH-9	Small observation plot	Cd. Obregon	2010	1717	263	45	100	76	173	45	87
DTH-10	Agua Fria	Agua Fria	2010	1717	261	49	81	93	125	87	87

Environment code of 12 combinations of sites in Mexico, agronomic management, and year for two wheat traits (grain yield, GY, and days to heading, DTH) from [11]. Number of markers, total number of individuals, number of individuals in the upper 15 and 30 % classes, in the middle 40 and 70 % classes, and in the lower 15 and 30 % classes from the empirical cumulative distribution

### Transforming phenotypic responses into three or two classes

The continuous phenotypic responses *y*_*i*_ for each stratified random partition in the datasets were grouped into three classes (upper, middle and lower), based on 15–85 % and 30–70 % percentiles of the response of each trait analyzed. For example, for 15–85 % percentiles, the quantiles *q*_0.15_ and *q*_0.85_ were used to split *y*_*i*_ into three classes: *y*_*i*_ ∈ upper class, if *y*_*i*_ > *q*_0.85_; *y*_*i*_ ∈ middle class, if *q*_0.15_ < *y*_*i*_ ≤ *q*_0.85_; and, *y*_*i*_ ∈ lower class; *if y*_*i*_ ≤ *q*_0.15_ A similar rule was applied to split y_*i*_ into three classes with 30-70 % percentiles.

For the two species, the target classes were the upper 15 and 30 % classes (GY for maize and wheat); the middle 40 and 70 % classes (ASI for maize), and the lower 15 and 30 % classes (FFL, MFL, and GLS for maize and DTH for wheat).

Comparison of prediction accuracy of PNN based on two or three classes was performed only on the wheat datasets to simplify computations. Firstly, the phenotypic responses *y*_*i*_ for each stratified random partition of the wheat datasets were grouped into two classes from the datasets previously grouped into three classes. The upper 15 % of the binary class was defined by using the upper 15 % of the trichotomous classes, and the lower class was the sum of the middle and lower classes of the trichotomous classes; a similar strategy was applied for the lower 15 % of the binary class. The same random partitions (training, testing sets) were used when comparing PNN with two classes versus PNN with three classes. Partitions of the wheat datasets into two classes for GY and DTH are shown in Table 3.

**Table 3:** Wheat datasets – two classes

				Number of individuals		Number of individuals	
Data set	Agronomic management	Site in Mexico	Year	Upper	Lower	Upper	Lower
				15 %	85 %	30 %	70 %
GY-1	Drought-bed	Cd. Obregon	2009	46	260	92	214
GY-2	Drought-bed	Cd. Obregon	2010	47	259	92	214
GY-3	Drought-flat	Cd. Obregon	2010	39	224	80	183
GY-4	Full irrigation-bed	Cd. Obregon	2009	46	258	92	212
GY-5	Full irrigation-bed	Cd. Obregon	2010	46	260	94	212
GY-6	Heat-bed	Cd. Obregon	2010	46	260	94	212
GY-7	Full irrigation-flat-borders	Cd. Obregon	2010	39	224	79	184
				Lower	Upper	Lower	Upper
				15 %	85 %	30 %	70 %
DTH-1	Drought-bed	Cd. Obregon	2009	53	253	100	206
DTH-2	Drought-bed	Cd. Obregon	2010	50	256	93	213
DTH-3	Drought-flat	Cd. Obregon	2010	40	223	86	177
DTH-4	Full irrigation-bed	Cd. Obregon	2009	59	247	107	199
DTH-5	Full irrigation-bed	Cd. Obregon	2010	47	259	101	205
DTH-6	Toluca	Toluca	2009	122	184	122	184
DTH-7	El Batan	El Batan	2009	66	240	104	202
DTH-8	Small observation plot	Cd. Obregon	2009	58	243	101	200
DTH-9	Small observation plot	Cd. Obregon	2010	45	218	100	163
DTH-10	Agua Fria	Agua Fria	2010	49	212	81	160

Environment code of 12 combinations of sites in Mexico, agronomic management, and year for two wheat traits (grain yield, GY, and days to heading, DTH) from [11]. Number of markers, total number of individuals, number of individuals in the upper 15 and 30 % classes, and in the lower 85 and 70 % classes

### Multilayer perceptron neural network (MLP) classifier

An MLP can be trained to classify items into *S* different disjoint classes. Each target class *c*_*i*_ is transformed into a target vector ***c***_*i*_ of zeroes except for a 1 in element *k*, (*k* = 1, …, *S*) the class to be represented. We arranged a set of *n* input vectors **x**_*i*_ into a matrix **X** of dimension *n* × *p*. Then we arranged the *n* target vectors *c*_*i*_ into a matrix **C** of dimension *S* × *n*. The rows of **X** correspond to columns of **C**, individual-by-individual. Statistical learning is inferred from the data only, with no assumption about the joint distribution of inputs and outcomes. This gives MLP great flexibility for capturing complex patterns frequently found in plant breeding [26].

**Fig. 1:**
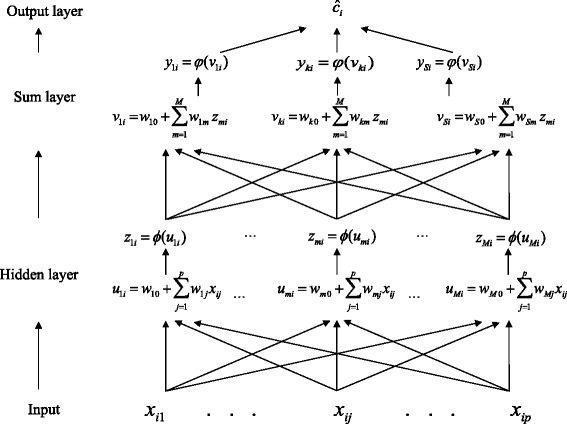
Architecture of classifier MLP with the input (markers) layer, hidden layer, and sum-output layer

We begin by describing a standard MLP for a categorical response (PNN is introduced subsequently). MLP is an NN that can be thought of as a two-stage regression (e.g., [18]). In the first stage (hidden layer), *M* data-derived basis functions, {*z*_*m*_}_*m* = 1_^*m* = *M*^ are inferred; in the second stage (the output layer has *S* neurons, *S* classes), each neuron’s output is computed on the basis functions inferred in the hidden layer using a nonlinear transfer function (Fig. 1).

In the hidden layer, one data-derived predictor is inferred at each of *M* neurons. These data-derived predictors are computed by first inferring a score (*u*_*mi*_), which is a linear combination of the input weights and the input markers plus a bias (intercept) term. Subsequently, this score is transformed using a nonlinear transfer function, *φ*(⋅), that is, *z*_*mi*_ = *φ*(*w*_*mo*_ + ∑_*j* = 1_^*p*^*w*_*mj*_*x*_*ij*_), where *w*_*mo*_ is the bias term, and *W*_*m*_ = {*W*_*mj*_}_*m* = 1; *j* = 1_^*m* = *M*; *j* = *p*^ is an input weight matrix. The transfer function maps from a score defined in the real line onto the interval [−1, 1] (e.g., a hyperbolic tangent sigmoid transfer function is $tansig(u)=\frac{2}{\left(\left(1+ exp\left(-2\right)\right)-1\right)}$. Subsequently, in the output layer, phenotypes are regressed on the data-derived features, {*z*_*mi*_}_*i* = 1; *m* = 1_^*i* = *n*; *m* = *M*^, according to the model E(*y*_*ki*_|*parameters*) = *v*_*ki*_ = *w*_*ko*_ + ∑_*m* = 1_^*M*^*w*_*mi*_, where *φ*_*k*_(*v*_*ki*_) and *φ*_*k*_(.) is the *tansig* transfer function, *k* = 1, …, *S*. Finally, the predicted score vector **ĉ**_*i*_ = {*y*_*ki*_}_*k* = 1_^*k* = *S*^, and the predicted class *ĉ*_*i*_ is *ĉ*(**x**_*i*_) = arg *max*_1 ≤ *k* ≤ *S*_(*ĉ*_*k*_) are obtained.

Training of an MLP (given a fixed number of transfer functions in the hidden layer) involves estimating all of the classifier’s parameters by means of an iterative backpropagation error algorithm, based on the scaled conjugate gradient algorithm described by [27]. To improve the generalization capacity of MLP, an early stopping ensemble strategy can be applied [28]; early stopping effects non-Bayesian shrinkage of coefficients. In this approach, we divided the available data into three subsets. The first subset is the training set, used for computing the gradient and updating network weights and biases. The second subset is the validation set, where the error in the set is monitored during the training process. The validation error normally decreases during the initial training phase, as does the training set error. However, when the network begins to over-fit the data, the error in the validation set typically begins to rise. When the validation error increases at some point in the iteration, the training is stopped, and the weights and biases at the minimum validation error are returned. The third subset is used as testing set.

The performance function to optimize an MLP is usually the mean squared error (*mse*), which is the average squared error between the predicted classes **Ĉ** and the target classes **C. Ĉ** is also a matrix of dimension *S* × *n*, where each column contains values in the [0,1] range. The index of the largest element in the column indicates which of the *S* classes that vector represents.

### Probabilistic neural network (PNN) classifier

**Fig. 2:**
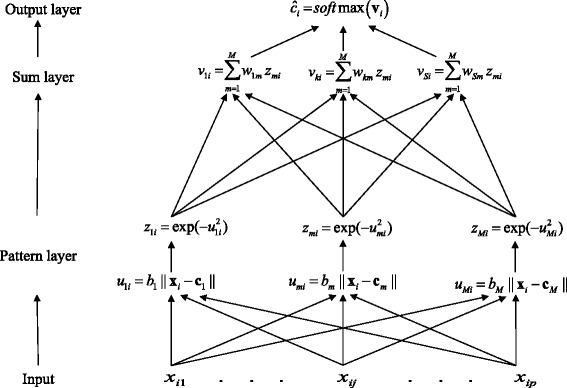
Architecture of classifier PNN with the input (markers) layer, pattern layer, and sum-output layer

The architecture of a PNN is similar to that of a radial basis function NN [8]; a PNN has two layers, the pattern layer and the summation-output layer, as illustrated in Fig. 2. The pattern layer computes distances (using a Gaussian radial basis function (RBF)) between the input vector **x**_*i*_ and the training (centers) input vectors **c**_*m*_ ∈ *ℝ*^*p*^; *m* = 1, …, *M* neurons (*M = n* individuals of the input data set) and returns an output vector **u**_*i*_ ∈ *ℝ*^*M*^ whose elements *u*_*mi*_ = *b*_*m*_‖**x**_*i*_ − ***c***_*m*_‖, where ${b}_m=\frac{\sqrt{\left(-\mathrm{In}(0.5)\right)}}{h}$ is a weight and *h* is the width of the Gaussian RBF, indicating how close the input vector **x**_*i*_ is to **c**_*m*_ [22]. Then each *u*_*mi*_ is transformed into a vector **z**_*i*_ ∈ *ℝ*^*M*^, whose elements are defined by the Gaussian operation *z*_*mi*_ = *exp*(−*u*_*mi*_^2^). The summation-output layer sums these contributions for each class *k*, that is, *v*_*ki*_ = ∑_*m* = 1_^*M*^*w*_*km*_*z*_*mi*_, where *w*_*km*_ are weights obtained from the target classes **C** matrix, to generate a vector of probabilities **ĉ**_*i*_ = *softmax*(**v**_*i*_) of dimension *S* × 1 as its net output, where the *softmax* transfer function *σ*(.) is given by

$$ \sigma \left({\mathbf{v}}_i\right)=\frac{exp\left({v}_k\right)}{{\displaystyle {\sum}_{j=1}^S exp\left({v}_j\right)}};\kern0.5em for\kern0.5em k=1,\dots, S\kern0.5em \mathrm{classes} $$

where **v**_*i*_ is a target vector of dimension *S* × 1 with elements *v*_*k*_. The *softmax* transfer function on the summation-output layer transforms the outputs of processing units for each *k* class in the interval [0,1].

The pattern layer of a PNN is a neural representation of a Bayes classifier, where the class density functions are approximated using a windows Parzen estimator [29]. The standard training method for a PNN (given a value of *h* for the Gaussian RBFs) requires a single pass over all the **x**_*i*_ markers of the training set. For this reason, PNN requires short training time and produce as output (***ĉ***_*i*_), posterior probabilities of class membership.

### Criteria for assessing classifier prediction accuracy

The prediction accuracy of MLP and PNN was evaluated using a cross-validation procedure. For each data set, 50 random partitions stratified by classes were generated. Each partition randomly assigned 90 % of the data to the training set and the remaining 10 % to the testing set. We used stratified sampling by class to make sure there were no empty classes in the training and testing sets. For each data set, partition index matrices PINDX(*n*, 50) were generated, where *n* is the number of individuals in each data set analyzed; PINDX(i,j) has a value equal to 1 (training) or 2 (testing) for the *i*^th^ individual in the *j*^th^ partition. Each model was trained and evaluated with the same pair of training and testing sets of each partition. For MLP the training sets defined in PINDX(*n*, 50) were subdivided by stratified random sampling by class into two disjoint sets, one for training (88 %) and another for validation (12 %); this was done with the objective of applying the training early stopping ensemble strategy[28]. For each random partition, ten replications (random seeds) were used to evaluate the performance of MLP.

Two performance measures for assessing prediction accuracy of the two classifiers (averaged across 50 random partitions) were used: (1) the area under the receiver operating characteristic curve (*AUC*), and (2) the area under the precision-recall curve (*AUCpr*), or average precision.

For GY in both species, models were trained to maximize the *AUC* of the upper class; for FL, GLS, and DTH, models were trained to maximize the *AUC* of the lower class; for ASI, the target value is zero (perfect synchrony between anthesis and silking interval), models were trained to maximize the *AUC* of the middle class.

### The area under the receiver operating characteristic curve (*AUC*)

Rather than computing the recall (*R*) [also called sensitivity or true positive rate (*tpr*)] and the false positive rate (*fpr*) for a fixed threshold τ, a set of thresholds was defined and then *tpr* vs *fpr*(*R* vs *f pr*) was plotted as an implicit function of τ; this is called an ROC curve.

The recall or sensitivity is $R=\frac{tp}{tp+fn},$ where *tp* is the number of positives predicted as positives and *fn* is the number of positives predicted as negatives. This measure evaluates the number of individuals that are correctly classified as a proportion of all the observed individuals in the target class. $fpr=\frac{fp}{fp+tn},$ where *fp* is the number of negatives predicted as positives and *tn* is the number of negatives predicted as negatives (Table 4).

**Table 4:** Description of a confusion matrix for binary classes with observed values and classifier predicted values

		Classifier predicted value		Sum
		1	0	
Observed	1	*tp*	*fn* (Type II error)	*tp + fn*
	0	*fp* (Type I error)	*tn*	*fp* + *tn*
Sum		*tp* + *fp*	*fn* + *tn*	*n*

*tp* true positive, *fp* false positive, *fn* false negative, *tn* true negative, *n* total number of individuals

To compare the performance of classifiers, the receiver operating characteristic curve **(**ROC) has to be reduced to a single scalar value representing the expected performance. A common method is to compute the area under the ROC curve (*AUC*), which produces a value between 0 and 1. If *AUC*(*a*) > *AUC*(*b*), then classifier *a* has a better average performance than classifier *b. AUC* can be interpreted as the probability that a randomly chosen individual is ranked as more likely to be of the target class than a randomly chosen individual of another class. The ROC graphs are a useful tool for visualizing the performance of the classifiers because they provide a richer measure of classification performance than other scalar measures [30].

### The area under the precision-recall curve (*AUCpr*)

A precision-recall curve is a plot of precision (*P*) vs *R* for a set of thresholds τ. $P=\frac{tp}{tp+fp}$ is defined as the fraction of positives predicted as positives with respect to all predicted positives (Table 4). Thus *P* measures the fraction of the predicted positives that is really positive, while *R* measures the fraction of the predictive positives that was in fact detected. This curve is summarized as a single number using the average precision (*AUCpr*), which approximates the area under the precision-recall curve [31]. This measure is recommended for classes of different sizes; upper or lower classes of 15 % had a lower number of individuals than the corresponding upper or lower classes of 85 %. *AUC* is commonly used to present results of binary decision problems in machine learning algorithms. However, when dealing with unbalanced classes, *AUCpr* curves give a more informative idea of a machine learning algorithm than *AUC* [32, 33].

### Software

Scripts for fitting models and performing cross-validations were written in MATLAB r2010b. All the analyses were performed in a Linux Workstation.

## Results and discussion

**Fig. 3:**
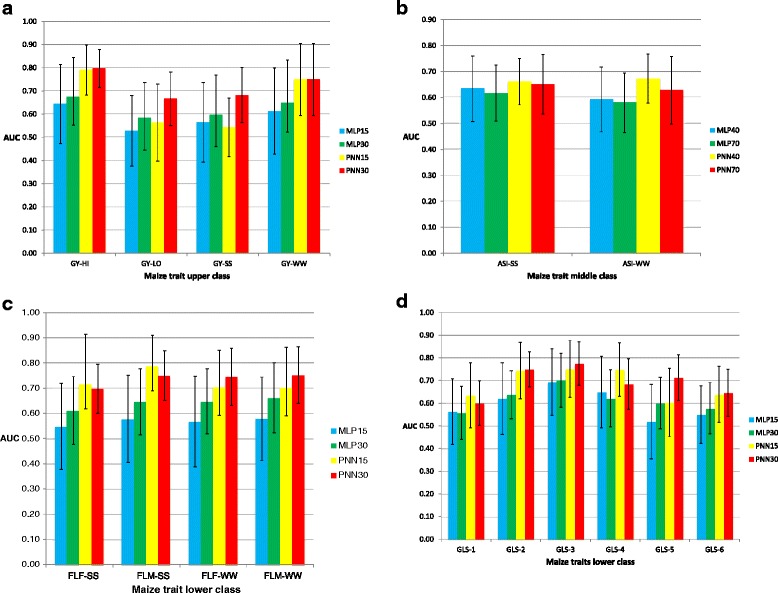
Histograms of the *AUC* criterion and their standard deviation (error bars) for the maize datasets. **a** grain yield (GY) under optimal conditions (HI and WW) and stress conditions (LO and SS) of classifiers MLP and PNN in the upper 15 and 30 % classes; **b** anthesis-silking interval (ASI) under optimal conditions (WW) and drought stress conditions (SS) of MLP and PNN of the middle 40 and 70 % classes; **c** female flowering time (FFL), male flowering time (MFL) under optimal well-watered (WW) conditions and drought stress conditions (SS) of MLP and PNN of the lower 15 and 30 % classes; **d** gray leaf spot resistance (GLS) in 6 environments (1–6) of MLP and PNN of the lower 15 and 30 % classes

**Fig. 4:**
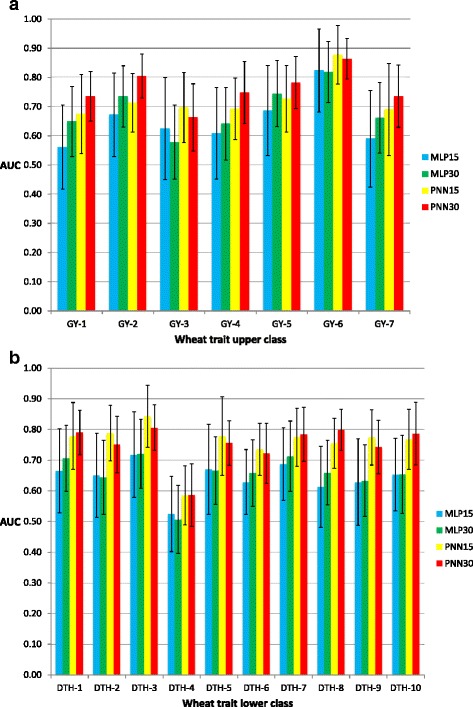
Histograms of the *AUC* criterion and their standard deviation (error bars) for the wheat datasets. **a** grain yield (GY) in seven environments (1–7) of classifiers MLP and PNN of the upper 15 and 30 % classes; **b** days to heading (DTH) in ten environments (1–10) of MLP and PNN in the lower 15 and 30 % classes

Results of the value of *AUC* for classifiers MLP and PNN in each trait-environment combination are depicted in histograms in Fig. 3a–d (maize datasets) and Fig. 4a–b (wheat datasets) for the traits selected in the upper and lower (15 and 30 %) and middle (40 and 70 %) classes, respectively.

The first clear trend using the *AUC* criterion is that PNN outperformed MLP for most of the individuals in the upper, middle and lower classes. Depending on the trait-environment combination, the PNN30% or PNN15% upper and lower and the PNN40% and PNN70% middle were usually larger than those of MLP; the only exception was PNN15% for GY-SS (Fig. 3a), which was lower than MLP15% (Additional file 1: Table S1).

We also describe *AUC* and *AUCpr* results of comparing the performance of PNN for wheat trait-environment combinations using two or three classes.

### Comparing classifiers to select individuals in the upper, middle and lower classes in the maize datasets

#### Upper classes (15 and 30 %)

Results of the prediction accuracy criterion *AUC* of the two classifiers MLP and PNN for traits selected in the 15 and 30 % upper classes for GY under the different environmental conditions are reported in Fig. 3a. PNN was more accurate than MLP in the upper 30 % class, for assigning individuals based on GY under stress conditions. Additional file 1: Table S1 shows the results based on the *AUC* criterion for the upper, middle and lower classes.

When using the *AUCpr* criterion, which relates *P* and *R* for the upper class, PNN outperformed MLP, which is clearly shown in Table 5 (as shown for the *AUC* criterion in Fig. 3a). Also, *AUCpr* for PNN30% was always better than PNN15% for all the traits in the upper class. These results lead to the conclusion that PNN was more accurate than MLP for assigning maize lines to the correct upper class for GY under WW and SS conditions. Also under the *AUC* criterion, PNN30% was similar to PNN15% for GY-HI and GY-WW, but better than PNN15% for GY-LO and GY-SS. Under the criterion *AUCpr*, PNN30% was always better than PNN15% for all GY.

**Table 5:** Maize datasets

Upper class								
	MLP15%		PNN15%		MLP30%		PNN30%	
GY-HI	0.235	(0.126)	**0.306**	(0.118)	0.429	(0.108)	**0.509**	(0.102)
GY-LO	0.168	(0.065)	**0.188**	(0.076)	0.358	(0.107)	**0.408**	(0.107)
GY-SS	0.199	(0.093)	**0.204**	(0.110)	0.363	(0.111)	**0.453**	(0.119)
GY-WW	0.239	(0.131)	**0.382**	(0.175)	0.410	(0.117)	**0.477**	(0.111)
Middle class								
	MLP40%		PNN40%		MLP70%		PNN70%	
ASI-SS	0.465	(0.096)	**0.495**	(0.092)	0.724	(0.076)	**0.746**	(0.074)
ASI-WW	0.436	(0.091)	**0.481**	(0.088)	0.706	(0.072)	**0.722**	(0.084)
Lower class								
	MLP15%		PNN15%		MLP30%		PNN30%	
FFL-SS	0.185	(0.087)	**0.288**	(0.137)	0.383	(0.106)	**0.465**	(0.096)
MFL-SS	0.205	(0.101)	**0.343**	(0.149)	0.421	(0.119)	**0.499**	(0.112)
FFL-WW	0.197	(0.102)	**0.298**	(0.161)	0.413	(0.120)	**0.506**	(0.133)
MFL-WW	0.199	(0.094)	**0.288**	(0.155)	0.437	(0.133)	**0.516**	(0.139)
GLS-1	0.269	(0.096)	**0.338**	(0.135)	0.476	(0.096)	**0.526**	(0.092)
GLS-2	0.320	(0.140)	**0.447**	(0.157)	0.524	(0.101)	**0.642**	(0.093)
GLS-3	0.372	(0.138)	**0.456**	(0.149)	0.496	(0.128)	**0.589**	(0.116)
GLS-4	0.350	(0.135)	**0.487**	(0.147)	0.439	(0.110)	**0.512**	(0.111)
GLS-5	0.161	(0.072)	**0.208**	(0.107)	0.429	(0.098)	**0.538**	(0.118)
GLS-6	0.320	(0.091)	**0.400**	(0.109)	0.431	(0.094)	**0.491**	(0.098)

Mean values of the area under the precision-recall curve *AUCpr AUCpr* (standard deviation in parentheses) of 50 random partitions for 15 and 30 % upper classes for grain yield (GY) in four environments (HI, LO, SS, and WW), for 40 and 70 % middle class for anthesis-silking interval (ASI) in two environments (SS and WW), and for 15 and 30 % lower classes for four traits, female flowering (FFL) and male flowering (MFL) in two environments (SS and WW); for gray leaf spot resistance (GLS) in six environments (1–6) and for classifiers MLP and PNN. Numbers in bold are the highest *AUCpr* values between MLP and PNN for 15 and 30 %

#### Middle classes (40 and 70 %)

Concerning the *AUC* criterion for the middle class based on ASI-SS and ASI-WW, Fig. 3b shows a slight superiority of PNN over MLP for both 40 and 70 %; however, PNN40% was, on average, slightly better than PNN70%. On the other hand, results using the *AUCpr* criterion also show a slight superiority of PNN over MLP for MLP40% for ASI-SS and MLP70% for both ASI-SS and ASI-WW (Table 5). For this middle class, the *AUCpr* results favored PNN as a better predictor than MLP for assigning maize lines to the correct middle class.

#### Lower classes (15 and 30 %)

For the lower class, Fig. 3c for FL and Fig. 3d for GLS (both traits in different environments) show a clear superiority in terms of the *AUC* criterion of PNN over MLP for both lower classes. The better prediction accuracy of classifier PNN is reflected in *AUCpr* prediction accuracy, where PNN outperformed MLP for both lower classes, and PNN30% was higher than PNN15% for all 10 traits (Table 5).

### Comparing classifiers for selecting individuals in the upper and lower classes in the wheat datasets

#### Upper classes (15 and 30 %)

Results of *AUC* for GY that were selected in the upper 15 and 30 % classes are presented in Fig. 4a and in Additional file 2: Table S2. PNN outperformed MLP for both upper classes for all GY. PNN30% gave better prediction accuracy than PNN15% in most traits, with the exception of GY-3 and GY-6, where PNN15% had better prediction than PNN30%.

Criterion *AUCpr* showed that PNN was better than MLP for both upper classes; PNN appeared as the best class predictive models in all GY traits. Furthermore, under the *AUCpr* criterion, PNN30% was higher than PNN15% in all wheat GY traits (Table 6). In summary, results of the upper 15 and 30 % classes show that PNN was a more accurate predictor than MLP when using the *AUC* and *AUCpr* criteria.

**Table 6:** Wheat datasets

	MLP15%		PNN15%		MLP30%		PNN30%	
Upper class								
GY-1	0.204	(0.084)	**0.288**	(0.140)	0.406	(0.113)	**0.475**	(0.102)
GY-2	0.270	(0.108)	**0.307**	(0.111)	0.485	(0.113)	**0.567**	(0.116)
GY-3	0.227	(0.114)	**0.268**	(0.108)	0.366	(0.100)	**0.453**	(0.118)
GY-4	0.242	(0.110)	**0.325**	(0.118)	0.409	(0.107)	**0.518**	(0.115)
GY-5	0.284	(0.115)	**0.326**	(0.142)	0.505	(0.116)	**0.550**	(0.107)
GY-6	0.504	(0.172)	**0.561**	(0.157)	0.637	(0.115)	**0.701**	(0.083)
GY-7	0.199	(0.091)	**0.290**	(0.117)	0.423	(0.114)	**0.529**	(0.115)
Lower class								
DTH-1	0.304	(0.113)	**0.414**	(0.124)	0.522	(0.107)	**0.630**	(0.091)
DTH-2	0.297	(0.117)	**0.429**	(0.132)	0.433	(0.110)	**0.521**	(0.104)
DTH-3	0.364	(0.149)	**0.511**	(0.151)	0.547	(0.115)	**0.650**	(0.095)
DTH-4	0.254	(0.077)	**0.298**	(0.089)	0.297	(0.070)	**0.363**	(0.097)
DTH-5	0.275	(0.131)	**0.384**	(0.164)	0.440	(0.104)	**0.546**	(0.087)
DTH-6	0.380	(0.091)	**0.467**	(0.094)	0.465	(0.099)	**0.520**	(0.112)
DTH-7	0.368	(0.114)	**0.482**	(0.113)	0.521	(0.124)	**0.591**	(0.115)
DTH-8	0.264	(0.097)	**0.382**	(0.103)	0.452	(0.102)	**0.599**	(0.095)
DTH-9	0.261	(0.103)	**0.367**	(0.112)	0.416	(0.099)	**0.535**	(0.107)
DTH-10	0.447	(0.109)	**0.553**	(0.114)	0.462	(0.112)	**0.578**	(0.124)

Mean values of the area under the precision-recall curve *AUCpr* (standard deviation in parentheses) of 50 random partitions for the 15 and 30 % upper classes for grain yield (GY) in 7 environments (1–7) and 15 and 30 % lower classes for days to heading (DTH) in 10 environments (1–10) for classifiers MLP and PNN. Numbers in bold are the highest *AUCpr* values between MLP and PNN for 15 and 30 %

#### Lower classes (15 and 30 %)

For the lower classes involving wheat DTH, *AUC* of PNN was higher than MLP for both 15 and 30 % percentiles and all traits (Fig. 4b). In five instances (DTH-2, DTH-3, DTH-5, DTH-6 and DTH-9), the PNN15% model was slightly more accurate than PNN30% when classifying individuals in this lower class.

The best performance of PNN was reflected in the prediction accuracy given by the *AUCpr* criterion, where PNN was better than MLP in both lower classes for all DTH traits. Likewise, PNN30% was always higher than PNN15% (Table 6).

### Prediction accuracy of PNN classifier with two and three classes in the wheat datasets

This section compares the performance of PNN in the upper and lower (15 and 30 %) classes for wheat GY and DTH traits, when two and three classes are formed and evaluated using the *AUC* (Table 7) and *AUCpr* (Table 8) criteria. For the *AUC* criterion, PNN with three classes was slightly better than PNN with two classes for most traits in the upper and lower 15 and 30 % classes (Table 7). For the *AUCpr* criterion, results were not as clear as for *AUC*; however, PNN with three classes was globally better than PNN with two classes (Table 8).

**Table 7:** Wheat datasets

	PNN15% (two classes)		PNN15% (three classes)		PNN30% (two classes)		PNN30% (three classes)	
Upper class								
GY-1	0.658	(0.140)	**0.675**	(0.135)	0.708	(0.082)	**0.735**	(0.085)
GY-2	0.691	(0.091)	**0.713**	(0.100)	0.765	(0.081)	**0.805**	(0.076)
GY-3	0.694	(0.123)	**0.697**	(0.120)	**0.664**	(0.115)	0.663	(0.115)
GY-4	0.674	(0.120)	**0.693**	(0.105)	0.701	(0.112)	**0.748**	(0.107)
GY-5	0.710	(0.123)	**0.727**	(0.115)	0.775	(0.083)	**0.782**	(0.089)
GY-6	**0.880**	(0.097)	0.878	(0.100)	0.830	(0.075)	**0.864**	(0.070)
GY-7	0.649	(0.160)	**0.690**	(0.158)	0.708	(0.116)	**0.736**	(0.106)
Lower class								
DTH-1	0.724	(0.112)	**0.779**	(0.109)	**0.791**	(0.074)	**0.791**	(0.072)
DTH-2	0.773	(0.094)	**0.789**	(0.090)	**0.763**	(0.100)	0.751	(0.092)
DTH-3	0.840	(0.100)	**0.843**	(0.101)	0.802	(0.074)	**0.806**	(0.074)
DTH-4	0.584	(0.098)	**0.585**	(0.097)	0.568	(0.094)	**0.587**	(0.102)
DTH-5	0.763	(0.121)	**0.779**	(0.128)	0.754	(0.075)	**0.756**	(0.072)
DTH-6	0.708	(0.086)	**0.736**	(0.085)	**0.722**	(0.097)	**0.722**	(0.098)
DTH-7	0.765	(0.096)	**0.775**	(0.095)	0.775	(0.097)	**0.785**	(0.088)
DTH-8	0.750	(0.080)	**0.755**	(0.082)	**0.803**	(0.065)	0.799	(0.067)
DTH-9	0.764	(0.105)	**0.774**	(0.090)	0.736	(0.088)	**0.743**	(0.087)
DTH-10	0.763	(0.763)	**0.768**	(0.098)	0.774	(0.094)	**0.787**	(0.102)

Mean values of the area under the ROC curve *AUC* (standard deviation in parentheses) of 50 random partitions for the 15 and 30 % upper class for grain yield (GY) in 7 environments (1–7) and for 15 and 30 % lower class for days to heading (DTH) for classifier PNN with two and three classes. Numbers in bold are the highest AUC values

**Table 8:** Wheat datasets

	PNN15% (two classes)		PNN15% (three classes)		PNN30% (two classes)		PNN30% (three classes)	
Upper class								
GY-1	0.270	(0.134)	**0.288**	(0.140)	**0.499**	(0.118)	0.475	(0.102)
GY-2	**0.322**	(0.118)	0.307	(0.111)	0.538	(0.117)	**0.567**	(0.116)
GY-3	**0.310**	(0.138)	0.268	(0.108)	0.452	(0.117)	**0.453**	(0.118)
GY-4	0.319	(0.121)	**0.325**	(0.118)	0.482	(0.112)	**0.518**	(0.115)
GY-5	**0.333**	(0.161)	0.326	(0.142)	0.545	(0.104)	**0.550**	(0.107)
GY-6	**0.562**	(0.159)	0.561	(0.157)	0.668	(0.087)	**0.701**	(0.083)
GY-7	0.263	(0.124)	**0.290**	(0.117)	0.503	(0.117)	**0.529**	(0.115)
Lower class								
DTH-1	0.370	(0.114)	**0.414**	(0.124)	0.629	(0.091)	**0.630**	(0.091)
DTH-2	0.417	(0.134)	**0.429**	(0.132)	**0.548**	(0.116)	0.521	(0.104)
DTH-3	0.506	(0.158)	**0.511**	(0.151)	0.641	(0.093)	**0.650**	(0.095)
DTH-4	0.292	(0.090)	**0.298**	(0.089)	0.350	(0.094)	**0.363**	(0.097)
DTH-5	0.355	(0.158)	**0.384**	(0.164)	**0.551**	(0.091)	0.546	(0.087)
DTH-6	0.444	(0.087)	**0.467**	(0.094)	**0.530**	(0.119)	0.520	(0.112)
DTH-7	0.462	(0.116)	**0.482**	(0.113)	0.580	(0.122)	**0.591**	(0.115)
DTH-8	**0.387**	(0.104)	0.382	(0.103)	**0.603**	(0.089)	0.599	(0.095)
DTH-9	**0.376**	(0.138)	0.367	(0.112)	0.532	(0.105)	**0.535**	(0.107)
DTH-10	**0.557**	(0.112)	0.553	(0.114)	0.575	(0.117)	**0.578**	(0.124)

Mean values of the area under the precision-recall curve *AUCpr* (standard deviation in parentheses) of 50 random partitions for the 15 and 30 % upper classes for grain yield (GY) in 7 environments (1–7) and for 15 and 30 % lower classes for days to heading (DTH) for classifier PNN with two and three classes. Numbers in bold are the highest AUCpr values

In summary, results for the wheat datasets comparing the performance of PNN for selecting individuals in the lower and upper 15 and 30 % classes, based on the splitting of continuous traits into two or three classes, showed that for the lower 15 %, the performance of PNN with three classes was better than PNN with two classes (in seven of ten traits). However, PNN with two classes gave better predictions than PNN with three classes in the upper 15 % (four over seven traits). This is not the case when predicting individuals in the upper and lower 30 %, where PNN with three classes was a better predictor than PNN with two classes for most traits.

### ROC and precision-recall curves for the maize and wheat datasets

**Fig. 5:**
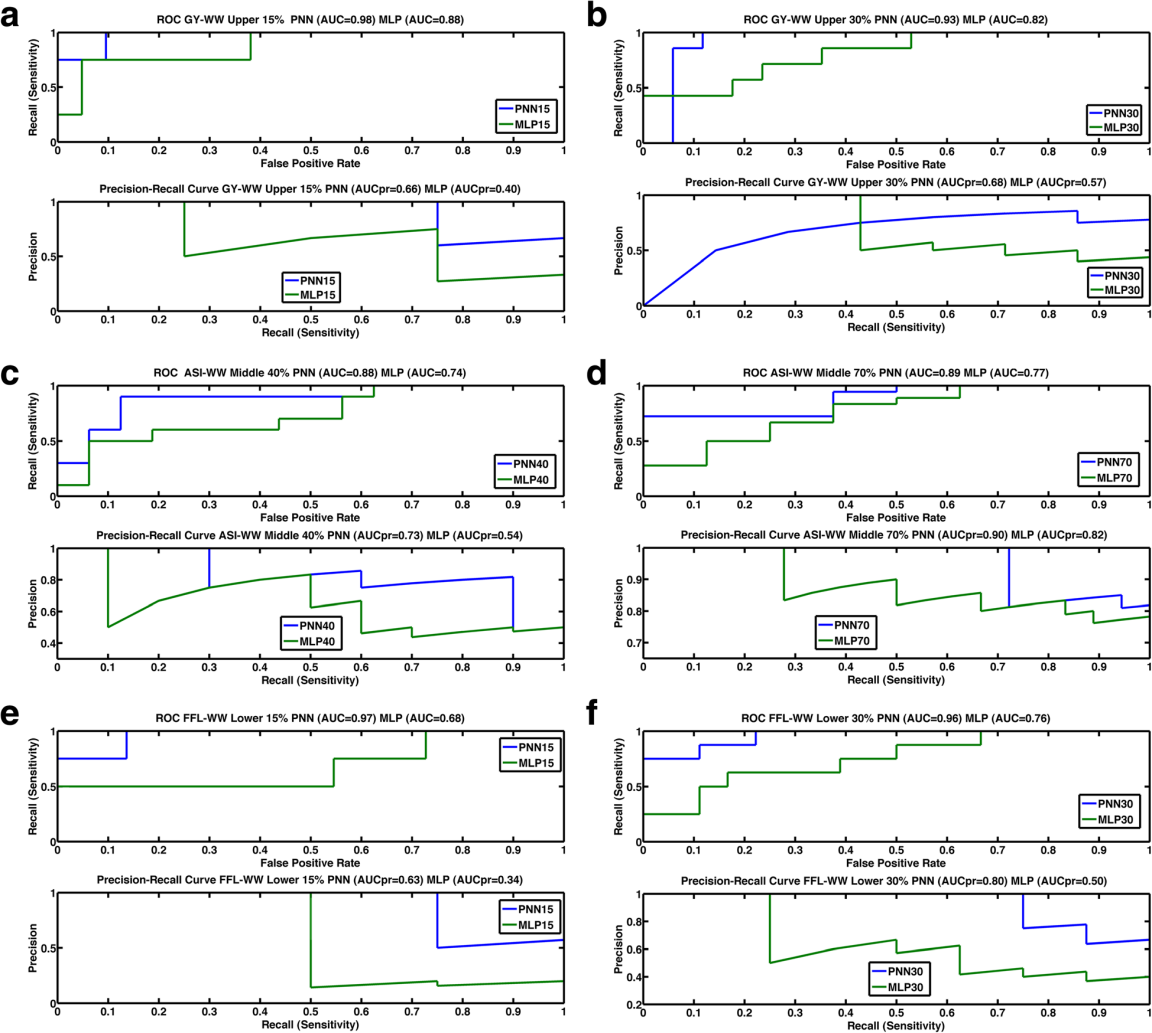
The upper curve is the ROC curve (*AUC*) with recall vs false positive rate. The lower curve is the precision-recall curve *AUCpr* with precision vs recall for the **a** upper 15 % class of grain yield under well-watered conditions (GY-WW) of classifiers MLP (green) and PNN (blue); **b** upper 30 % class of trait grain yield under well-watered conditions (GY-WW) of MLP (green) and PNN (blue); **c** middle 40 % class of trait anthesis-silking interval under well-watered conditions (ASI-WW) of MLP (green) and PNN (blue) and **d** middle 70 % class of trait anthesis-silking interval under well-watered conditions (ASI-WW) of MLP (green) and PNN (blue); **e** lower 15 % class of trait female flowering under well-watered conditions (FFL-WW) of MLP (green) and PNN (blue); **f** lower 30 % class of trait female flowering under well-watered conditions (FFL-WW) of MLP (green) and PNN (blue)

**Fig. 6:**
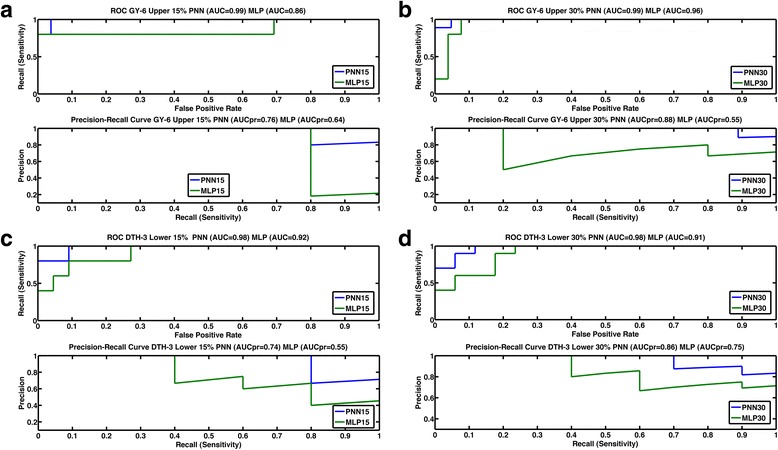
The upper curve is the ROC curve (*AUC*) with recall (sensitivity) vs false positive rate. The lower curve is the precision-recall curve *AUCpr* with precision vs recall for the **a** upper 15 % class of grain yield in environment 6 (GY-6) of classifiers MLP (green) and PNN (blue); **b** upper 30 % class of grain yield in environment 6 (GY-6) of MLP (green) and PNN (blue); **c** lower 15 % class of days to heading in environment 3 (DTH-3) of MLP (green) and PNN (blue); **d** lower 30 % class of days to heading in environment 3 (DTH-3) of MLP (green) and PNN (blue)

Some results of the ROC and precision-recall curves for various maize and wheat datasets for upper and lower 15 and 30 %, with the middle class in maize for 40 and 70 %, are displayed in a series of figures (for the maize datasets, Fig. 5a–f; for the wheat datasets, Fig. 6a–d). For the maize and wheat datasets, it is clear that the ROC curves of PNN for the upper and lower 15 and 30 % and the middle 40 and 70 % dominated the corresponding curves of MLP. Also, *AUC* values for PNN were always greater than those for MLP.

Furthermore, the *P* vs *R* graphs show that for all the maize and wheat datasets, PNN was better than MLP, indicating that the precision of PNN remains better than that of the MLP for all recall values. The precision of PNN started declining at higher values of R than the values of R for MLP.

## Discussion

### Accuracy of the MLP and PNN classifiers for selecting the best individuals

Genomic selection aims to accurately predict genetic values with genome-wide marker data using a three-step process: 1) model training and validation, 2) predicting genetic values, and 3) selecting based on these predictions [34].

We evaluated the performance of classifiers MLP and PNN for selecting the best individuals in maize and wheat datasets (Tables 1 and 2). Results indicated that, overall, PNN was more precise in identifying individuals in the correct class than MLP. Previous studies using RBFNN and Bayesian regularized NN on the same wheat datasets [8, 11] used in this study showed their prediction advantage over the linear parametric models for complex traits such as GY because these models can capture cryptic epistatic effects in gene × gene networks such as those usually present in wheat (e.g., additive × additive interactions). The good performance of PNN for selecting individuals in the correct classes may also be due to its ability for capturing small and complex interactions, while MLP may fail to do so.

The fact that these classifiers are trained to maximize the probability of membership of an individual to the target class, rather than searching for an overall performance, makes it attractive for applying these tools in GS. Results from MLP and PNN indicated that PNN was much more efficient in maximizing the probability of membership for the upper, middle, and lower classes than MLP.

From a practical genome-assisted plant breeding perspective, this study attempts to mimic the breeder’s decision, for example when selecting the upper 15 or 30 % class candidates for GY, or when selecting the lower 15 or 30 % class candidates for DTH, GLS or FL. In maize breeding, ASI synchrony close to zero is a crucial “middle class trait” under SS conditions because it will ensure selecting plants that will simultaneously produce pollen and silk; thus grains can be harvested. Therefore, PNN should help genomic-assisted breeding select appropriate candidates in each class of interest.

Breeding values have two main components, parental average (accounting for between family variation) and Mendelian sampling (accounting for within family variation). Genomic prediction should account for these two main components and try to control potential population structures that could modify prediction accuracy between the selected training and testing populations. An important practical question is how well PNN and MLP predict the breeding value of individuals between families and within families that were not phenotyped. Although the elite maize and wheat lines used in this study are not ideal as training sets, the cross-validation scheme used in this study (where 50 random partitions stratified by classes were generated for each data set) attempts to mimic the prediction of non phenotyped individuals belonging to different families (crosses) or to the same family. Although this cross-validation design may not have chosen individuals between and within families as precisely as they are in reality, it is likely that the 50 random partitions searched for all possible relationships between individuals in the training and testing sets such that some cross-validation partitions selected subsets of training data that had high correlations with the observed data, indicating a family relationship among individuals belonging to those training–testing subsets [11], whereas other random partitions chose subsets of training individuals that had no family relationship with those in the testing set, thus producing low correlations with the observed values. When applied to both classifiers, PNN consistently gave better average prediction accuracy (across the 50 random partitions) of the genetic values of the unobserved individuals than MLP in all 33 maize and wheat data sets.

### *AUC* and *AUCpr*

For both datasets, the results of the *AUCpr* criterion showed that the values of the upper and lower PNN30% were higher than those for the upper and lower PNN15%. Also, the values of the middle PNN70% were higher than those for the PNN40% (Tables 5 and 6). These results were similar but not equal to those found by *AUC* (which does not account for imbalances in the number of individuals comprising the upper, middle and lower classes) in several instances. PNN15% was superior to PNN30% in the maize data (e.g., ASI-SS, ASI-WW, FFL-SS, MFL-SS, GL-1, GLS-4) and the wheat data (e.g., DTH-2, DTH-3, DTH5, DTH-6, DTH-9). Prediction accuracy of individuals was clearly hampered under biotic stress in the maize data, which was also found by [6, 8, 11, 35].

Figures 5a–f and 6a–d showing the ROC curve clearly indicated the advantage of PNN over MLP. The *R* vs *fpr* graph indicates that, for most of the traits, the probability of correctly classifying an individual in the upper, lower or middle classes was very often 0.80 or higher, even with a small *fpr*. In most cases, at a value of *fpr* = 0, the probability of classifying an individual in the correct class was 0.80 or greater for PNN. For all traits, the *AUC* of PNN15% was always better than the *AUC* of MLP15% and the *AUC* of PNN30% was better than the *AUC* of MLP30%.

For the *AUCpr* curve, Figs. 5a–f and 6a–d indicate that, in most cases, PNN had higher precision than MLP at higher sensitivity values. This criterion also indicates the superior performance of PNN over MLP.

### Prediction accuracy for 30 vs 15 % classes with binary and trichotomous classes

Based on the *AUC* criterion, it is clear that PNN gave better prediction accuracy than MLP when assigning maize and wheat individuals to the classes of interest. Using the *AUCpr* criterion, the results were equally clear for the wheat and the maize datasets.

For the wheat datasets, the *AUC* criterion showed the superiority of PNN30% with three classes over PNN30% with two classes, as well as the superiority of PNN15% with three classes over PNN15% with two classes (Table 7). However, the differences given by the *AUC* criterion were not as marked as those shown by the *AUCpr* criterion. The *AUCpr* criterion applied with PNN shows that for the upper 15 % classes (GY traits), partitioning the data into two classes assigned more wheat lines to the correct observed classes than partitioning the data into three classes. However, for the lower 15 % classes (DTH traits) and for PNN 30 % upper and lower classes, results indicate that three classes gave better prediction than two classes (Table 8).

## Conclusions

We compared the performance of the multilayer perceptron (MLP) and the probabilistic neural network (PNN) classifiers for selecting the best individuals belonging to a class of interest (target class) in maize and wheat datasets using high-throughput molecular marker information (55 k and 1.4 k). PNN outperformed MLP in most of the datasets. The performance criteria used to judge the predictive accuracy of MLP and PNN for assigning individuals to the right observed class were the area under ROC curve, *AUC,* and the area under the precision-recall curve, *AUCpr*, PNN had better accuracy than MLP. In genomic selection, where p markers > > n individuals is the norm, PNN seems promising because of its better generalization capacity than MLP, and is faster than MLP in obtaining optimal solutions, thus presenting appealing computational advantages.

### Availability of supporting data

The 33 datasets (16 maize and 17 wheat trials) and the MATLAB scripts used in this work are available at http://hdl.handle.net/11529/10576.

## Additional files

Additional file 1: Table S1. Maize datasets. Mean values of the area under the ROC curve *AUC* (standard deviation in parentheses) of 50 random partitions for upper 15 and 30 % classes for grain yield (GY) in four environments (HI, LO, SS, and SS), for middle 40 and 70 % classes for anthesis-silking interval (ASI) in two environments (SS) and (WW), and for lower 15 and 30 % classes for four traits, female flowering (FFL) and male flowering (MFL) in two environments (WW and SS); for gray leaf spot resistance (GLS) in six environments and for both MLP and PNN classifiers. Numbers in bold are the highest *AUC* values between MLP and PNN for 15 and 30 %. (DOC 62 kb) Additional file 2 :Table S2. Wheat datasets. Mean values of the area under the ROC curve *AUC* (standard deviation in parentheses) of 50 random partitions for the upper 15 and 30 % classes for grain yield (GY) in seven environments (1-7) and for the lower 15 and 30 % classes for days to heading (DTH) in ten environments (1-10) for both MLP and PNN classifiers. Numbers in bold are the highest *AUC* values between MLP and PNN for 15 and 30 %. (DOC 59 kb)

## Abbreviations

ASI: anthesis-silking interval
*AUC*: area under the receiver operating characteristic curve
*AUCpr*: area under the precision-recall curve
DTH: days to heading
FFL: female flowering or days to silking
GLS: gray leaf spot
GS: genomic selection
GY: grain yield
HI: high yielding
LO: low yielding
MFL: male flowering or days to anthesis
MLP: multi-layer perceptron
NN: neural networks
*P*: Precision
PNN: probabilistic neural network
QTL: quantitative trait loci
*R*: recall or sensitivity
RBF: radial basis function
RBFNN: radial basis function neural network
RKHS: reproducing kernel Hilbert spaces
ROC: receiver operating characteristic curve
SS: severe drought stress
WW: well-watered
