# Supplementary material for: Genome-enabled prediction using probabilistic neural network classifiers
Source: BMC Genomics. 2016 Mar 9;17:208. doi: 10.1186/s12864-016-2553-1 (PMC4784384; doi:10.1186/s12864-016-2553-1)
Supplement: Additional file 1: Table S1. — Maize datasets. Mean values of the area under the ROC curve AUC (standard deviation in parentheses) of 50 random partitions for upper 15 and 30 % classes for grain yield (GY) in four environments (HI, LO, SS, and SS), for middle 40 and 70 % classes for anthesis-silking interval (ASI) in two environments (SS) and (WW), and for lower 15 and 30 % classes for four traits, female flowering (FFL) and male flowering (MFL) in two environments (WW and SS); for gray leaf spot resistance (GLS) in six environments and for both MLP and PNN classifiers. Numbers in bold are the highest AUC values between MLP and PNN for 15 and 30 %. (DOC 62 kb) [file 12864_2016_2553_MOESM1_ESM.doc]

| Supplemental Table S1. Maize datasets. Mean values of the area under the ROC curve 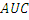 (standard deviation in parentheses) of 50 random partitions for upper 15 and 30% classes for grain yield (GY) in four environments (HI, LO, SS, and SS), for middle 40 and 70% classes for anthesis-silking interval (ASI) in two environments (SS) and (WW), and for lower 15 and 30% classes for four traits, female flowering (FFL) and male flowering (MFL) in two environments (WW and SS); for gray leaf spot resistance (GLS) in six environments and for both MLP and PNN classifiers. Numbers in bold are the highest 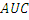 values between MLP and PNN for 15 and 30%. | | | | | | | | |
| --- | --- | --- | --- | --- | --- | --- | --- | --- |
| ---------------------------------------------Upper class-------------------------------------- | | | | | | | | |
|  | MLP15% | | PNN15% | | MLP30% | | PNN30% | |
| GY-HI | 0.643 | (0.171) | **0.790** | (0.108) | 0.674 | (0.121) | **0.796** | (0.081) |
| GY-LO | 0.527 | (0.152) | **0.564** | (0.166) | 0.585 | (0.141) | **0.666** | (0.115) |
| GY-SS | **0.565** | (0.171) | 0.543 | (0.126) | 0.598 | (0.139) | **0.682** | (0.119) |
| GY-WW | 0.613 | (0.186) | **0.749** | (0.156) | 0.648 | (0.125) | **0.749** | (0.156) |
| ----------------------------------------------Middle class------------------------------------- | | | | | | | | |
|  | MLP40% | | PNN40% | | MLP70% | | PNN70% | |
| ASI-SS | 0.634 | (0.108) | **0.661** | (0.089) | 0.617 | (0.126) | **0.651** | (0.115) |
| ASI-WW | 0.593 | (0.115) | **0.673** | (0.095) | 0.580 | (0.125) | **0.628** | (0.130) |
| ----------------------------------------------Lower class------------------------------------- | | | | | | | | |
|  | MLP15% | | PNN15% | | MLP30% | | PNN30% | |
| FFL-SS | 0.548 | (0.172) | **0.715** | (0.198) | 0.611 | (0.135) | **0.698** | (0.097) |
| MFL-SS | 0.578 | (0.173) | **0.788** | (0.123) | 0.646 | (0.131) | **0.750** | (0.098) |
| FFL-WW | 0.568 | (0.179) | **0.705** | (0.145) | 0.647 | (0.129) | **0.745** | (0.113) |
| MFL-WW | 0.579 | (0.165) | **0.703** | (0.160) | 0.662 | (0.139) | **0.753** | (0.112) |
| GLS-1 | 0.564 | (0.145) | **0.635** | (0.144) | 0.557 | (0.117) | **0.600** | (0.098) |
| GLS-2 | 0.620 | (0.158) | **0.743** | (0.124) | 0.637 | (0.105) | **0.749** | (0.078) |
| GLS-3 | 0.693 | (0.147) | **0.751** | (0.125) | 0.701 | (0.119) | **0.775** | (0.096) |
| GLS-4 | 0.649 | (0.157) | **0.748** | (0.118) | 0.622 | (0.126) | **0.684** | (0.111) |
| GLS-5 | 0.518 | (0.164) | **0.603** | (0.151) | 0.600 | (0.114) | **0.712** | (0.101) |
| GLS-6 | 0.549 | (0.127) | **0.639** | (0.124) | 0.577 | (0.114) | **0.646** | (0.104) |
