# Supplementary material for: Genome-enabled prediction using probabilistic neural network classifiers
Source: BMC Genomics. 2016 Mar 9;17:208. doi: 10.1186/s12864-016-2553-1 (PMC4784384; doi:10.1186/s12864-016-2553-1)
Supplement: Additional file 2 :Table S2. — Wheat datasets. Mean values of the area under the ROC curve AUC (standard deviation in parentheses) of 50 random partitions for the upper 15 and 30 % classes for grain yield (GY) in seven environments (1-7) and for the lower 15 and 30 % classes for days to heading (DTH) in ten environments (1-10) for both MLP and PNN classifiers. Numbers in bold are the highest AUC values between MLP and PNN for 15 and 30 %. (DOC 59 kb) [file 12864_2016_2553_MOESM2_ESM.doc]

| Supplemental Table S2. Wheat datasets. Mean values of the area under the ROC curve 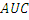(standard deviation in parentheses) of 50 random partitions for the upper 15 and 30% classes for grain yield (GY) in seven environments (1-7) and for the lower 15 and 30% classes for days to heading (DTH) in ten environments (1-10) for both MLP and PNN classifiers. Numbers in bold are the highest 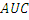 values between MLP and PNN for 15 and 30%. | | | | | | | | |
| --- | --- | --- | --- | --- | --- | --- | --- | --- |
|  | MLP15% | | PNN15% | | MLP30% | | PNN30% | |
| ---------------------------------------------Upper class-------------------------------------- | | | | | | | | |
| GY-1 | 0.561 | (0.145) | **0.675** | (0.135) | 0.649 | (0.120) | **0.735** | (0.085) |
| GY-2 | 0.672 | (0.143) | **0.713** | (0.100) | 0.735 | (0.105) | **0.805** | (0.076) |
| GY-3 | 0.625 | (0.175) | **0.697** | (0.120) | 0.578 | (0.126) | **0.663** | (0.115) |
| GY-4 | 0.609 | (0.157) | **0.693** | (0.105) | 0.641 | (0.124) | **0.748** | (0.107) |
| GY-5 | 0.686 | (0.155) | **0.727** | (0.115) | 0.744 | (0.113) | **0.782** | (0.089) |
| GY-6 | 0.824 | (0.143) | **0.878** | (0.100) | 0.819 | (0.105) | **0.864** | (0.070) |
| GY-7 | 0.590 | (0.165) | **0.690** | (0.158) | 0.662 | (0.121) | **0.736** | (0.106) |
| ------------------------------------------Lower class----------------------------------------- | | | | | | | | |
| DTH-1 | 0.665 | (0.136) | **0.779** | (0.109) | 0.706 | (0.108) | **0.791** | (0.072) |
| DTH-2 | 0.651 | (0.137) | **0.789** | (0.090) | 0.644 | (0.121) | **0.751** | (0.092) |
| DTH-3 | 0.718 | (0.139) | **0.843** | (0.101) | 0.720 | (0.112) | **0.806** | (0.074) |
| DTH-4 | 0.525 | (0.123) | **0.585** | (0.097) | 0.507 | (0.111) | **0.587** | (0.102) |
| DTH-5 | 0.670 | (0.147) | **0.779** | (0.128) | 0.666 | (0.110) | **0.756** | (0.072) |
| DTH-6 | 0.629 | (0.106) | **0.736** | (0.085) | 0.659 | (0.109) | **0.722** | (0.098) |
| DTH-7 | 0.687 | (0.118) | **0.775** | (0.095) | 0.713 | (0.115) | **0.785** | (0.088) |
| DTH-8 | 0.613 | (0.132) | **0.755** | (0.082) | 0.660 | (0.105) | **0.799** | (0.067) |
| DTH-9 | 0.629 | (0.141) | **0.774** | (0.090) | 0.633 | (0.117) | **0.743** | (0.087) |
| DTH-10 | 0.654 | (0.118) | **0.768** | (0.098) | 0.654 | (0.128) | **0.787** | (0.102) |
